# Circ_0000376 regulates miR-577/HK2/LDHA signaling pathway to promote the growth, invasion and glycolysis of osteosarcoma

**DOI:** 10.1186/s13018-023-04520-y

**Published:** 2024-01-13

**Authors:** Hongchun Dai, Guangming Yi, Dong Jiang, Yanmei Min, Zongwei Li

**Affiliations:** 1 Department of Oncology, The Third Hospital of Mianyang, Sichuan Mental Health Center, Mianyang, Sichuan China; 2 Department of Foot and Ankle Surgery, Mianyang Orthopedic Hospital, No.30, Nanhe Road, Fucheng District, Mianyang City, 621000 Sichuan China

**Keywords:** Osteosarcoma, circ_0000376, miR-577, HK2, LDHA

## Abstract

**Background:**

Many studies have confirmed that circular RNAs (circRNAs) mediate the malignant progression of various tumors including osteosarcoma (OS). Our study is to uncover novel molecular mechanisms by which circ_0000376 regulates OS progression.

**Methods:**

The expression of circ_0000376, microRNA (miR)-577, hexokinase 2 (HK2) and lactate dehydrogenase-A (LDHA) was determined by quantitative real-time PCR. OS cell proliferation, apoptosis and invasion were measured using cell counting kit 8 assay, colony formation assay, EdU assay, flow cytometry and transwell assay. Besides, cell glycolysis was assessed by testing glucose consumption, lactate production, and ATP/ADP ratios. Protein expression was examined by western blot analysis. The interaction between miR-577 and circ_0000376 or HK2/LADA was verified by dual-luciferase reporter assay. The role of circ_0000376 on OS tumor growth was explored by constructing mice xenograft models.

**Results:**

Circ_0000376 had been found to be upregulated in OS tissues and cells. Functional experiments revealed that circ_0000376 interference hindered OS cell growth, invasion and glycolysis. Circ_0000376 sponged miR-577 to reduce its expression. In rescue experiments, miR-577 inhibitor abolished the regulation of circ_0000376 knockdown on OS cell functions. MiR-577 could target HK2 and LDHA in OS cells. MiR-577 suppressed OS cell growth, invasion and glycolysis, and these effects were reversed by HK2 and LDHA overexpression. Also, HK2 and LDHA expression could be regulated by circ_0000376. In vivo experiments showed that circ_0000376 knockdown inhibited OS tumorigenesis.

**Conclusion:**

Circ_0000376 contributed to OS growth, invasion and glycolysis depending on the regulation of miR-577/HK2/LDHA axis, providing a potential target for OS treatment.

**Graphical Abstract:**

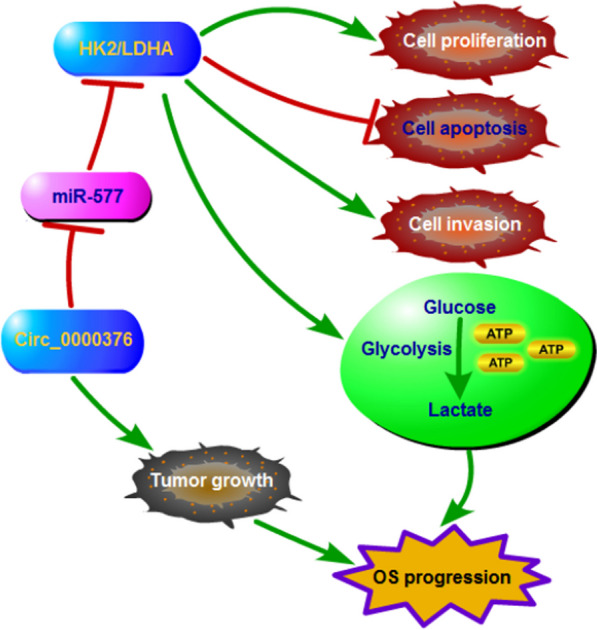

**Supplementary Information:**

The online version contains supplementary material available at 10.1186/s13018-023-04520-y.

## Highlights

- Circ_0000376 knockdown inhibits OS development and tumor growth.
- Circ_0000376 sponges miR-577.
- MiR-577 targets HK2 and LDHA.

**Supplementary Information:**

The online version contains supplementary material available at 10.1186/s13018-023-04520-y.

## Introduction

Osteosarcoma (OS) is the most common primary bone tumor in adolescents [1, 2]. The malignancy of OS is high, and most patients develop lung metastasis within one year, so the prognosis is poor [3, 4]. At present, surgery combined with chemotherapy drugs is still the main way of OS treatment, but the effect is limited [5, 6]. It is important to elucidate the underlying mechanisms affecting OS progression at the molecular level for developing potential therapeutic targets of OS.

Circular RNAs (circRNAs) are RNA molecules characterized by covalently closed loops and widely present in eukaryotes, which are mainly formed by back-splicing of exons or introns of genes [7, 8]. Mechanistically, circRNAs have been confirmed to act as microRNA (miRNA) sponges to mediate gene expression [9, 10]. A large amount of evidence shows that circRNA abnormal expression is often related to the occurrence of human diseases [11, 12]. Importantly, studies has confirmed that circRNA is associated with malignant progression of tumors, including OS [13, 14]. Studies had suggested that circTADA2A had an increasing effect on OS cell proliferation and metastasis, which was achieved via sponging miR-203a-3p to upregulate CREB3 [15]. Circ_0001721 was considered to be a potential target for OS treatment, which enhanced OS glycolysis, proliferation and metastasis through regulation of miR-372-3p/MAPK7 [16].

Circ_0000376 is located at chr12: 11199618-11248400 with 48,782 bp length and is derived from PRH1-PRR4 gene. In this study, we screened differentially expressed circRNA in OS tissues and normal tissues using GEO database, and pointed out that circ_0000376 was overexpressed in OS tissues. Previous studies had shown that decreased circ_0000376 expression could lead to decreased OS cell viability and metastasis ability [17]. Therefore, we have reason to believe that circ_0000376 may be a potential target for OS therapy. To further confirm this, we conducted this study and revealed a novel downstream miRNA/mRNA regulatory axis of circ_0000376.

## Materials and methods

### Samples collection

The OS tumor tissues and adjacent normal tissues were collected from 33 OS patients at The Third Hospital of Mianyang and stored at -80 °C. Written informed consent was signed from each patient, and our research was approved by The Third Hospital of Mianyang.

### Cell culture and transfection

OS cells (143B, HOS, MG63 and U2OS) and osteoblast cells (hFOB1.19) were bought from ATCC (Manassas, VA, USA) and cultured in DMEM medium (Solarbio, Beijing, China) containing 10% FBS and 1% penicillin–streptomycin. Circ_0000376 small interfering RNA (si-circ_0000376), pCD5 overexpression vector, lentivirus short hairpin RNA (sh-circ_0000376), miR-577 mimic, miR-577 inhibitor (anti-miR-577), pcDNA hexokinase 2 (HK2) overexpression vector, pcDNA lactate dehydrogenase-A (LDHA) overexpression vector, and negative controls were synthesized by RiboBio (Guangzhou, China). They were transfected into OS cells with Lipofectamine 3000 (Invitrogen, Carlsbad, CA, USA).

### Quantitative real-time PCR (qRT-PCR)

Total RNAs were isolated by TRIzol reagent (Invitrogen) and reverse-transcribed into cDNA using Reverse Transcription Kit (Takara, Dalian, China). PCR reaction was conducted with SYBR Green (Takara) and specific primers (Table 1). Relative expression was normalized by β-actin or U6 and expressed using 2^−ΔΔCT^ method. Also, RNA was treated with RNase R solution and then used for qRT-PCR.

**Table 1:** Primer sequences used for qRT-PCR

Name		Primers (5′-3′)
circ_0000376	Forward Reverse	TTTGGATGTGGAGGGGAATA GAGCCCAGGAGTTCCAGACT
miR-577	Forward Reverse	TGCGGTAGATAAAATATTGG CCAGTGCAGGGTCCGAGGT
LDHA	Forward Reverse	ATGGCAACTCTAAAGGATCAGC CCAACCCCAACAACTGTAATCT
HK2	Forward Reverse	GAGCCACCACTCACCCTACT CCAGGCATTCGGCAATGTG
GAPDH	Forward Reverse	CTCTGCTCCTCCTGTTCGAC CGACCAAATCCGTTGACTCC
β-actin	Forward Reverse	CTCCATCCTGGCCTCGCTGT GCTGTCACCTTCACCGTTCC
U6	Forward Reverse	CTCGCTTCGGCAGCACA AACGCTTCACGAATTTGCGT

### Cell proliferation detection

In cell counting kit 8 (CCK8) assay, OS cells seeded into 96-well plates were cultured for 48 h. CCK8 reagent (Beyotime, Shanghai, China) was added to each well. The absorbance at 450 nm was detected under microplate reader to measure cell viability.

In colony formation assay, OS cells seeded in 12-well plates were cultured for 2 weeks. After that, the colonies were fixed with paraformaldehyde and stained with crystal violet. The number of colonies was counted under microscope.

In EDU assay, OS cells seeded into 96-well plates were stained with EDU solution and DAPI solution (RiboBio). Fluorescence images were captured under fluorescence microscope, and EDU positive cell rate was calculated by ImageJ software.

### Flow cytometry

Annexin V-FITC Apoptosis Detection Kit (Beyotime) was used. OS cells suspended with binding buffer were stained with Annexin V-FITC and propidium iodide. Cell apoptosis rate was analyzed by flow cytometer and CellQuest software.

### Transwell assay

Transwell chamber pre-covered with Matrigel was used. Serum medium was added to the lower chamber, and OS cells suspended with DMEM medium were seeded into the upper chamber. 24 h later, the cells were fixed and stained. Under microscope, the number of invasive cells from 5 fields was counted.

### Cell glycolysis detection

After transfection, the supernatants of OS cells were collected for measuring the glucose consumption, lactate production and ATP/ADP level by Glucose Assay Kit, Lactate Assay Kit and ApoSENSOR ADP/ATP Ratio Assay (BioVision, Milpitas, CA, USA). The ECAR and OCR of cells were analyzed using XF96 Extracellular Flux analyzer (Seahorse Bioscience, Chicopee, MA, USA).

### Western blot (WB) analysis

RIPA buffer (Abcam, Cambridge, MA, USA) was used to obtain total protein. Protein samples were separated via SDS-PAGE gel and transferred onto PVDF membranes. Primary antibodies, including anti-CyclinD1 (1:200, ab16663), anti-MMP9 (1:1000, ab38898), anti-HK2 (1:10000, ab227198), anti-LDHA (1:5000, ab52488), and anti-β-actin (1:1000, ab8227), were used to incubate the membranes, which were then hatched with secondary antibody (1:50,000, ab205718). Protein bands were visualized using ECL reagent (Beyotime), and Image Lab software was used for gray scale analysis.

### Dual-luciferase reporter assay

The binding sequence and mutant sequence of miR-577 in circ_0000376, HK2 3’UTR or LDHA 3’UTR were designed and inserted into the pmirGLO reporter vector, generating the corresponding wild-type and mutant-type vectors. OS cells were co-transfected with the vectors and miRNA. Cells were then harvested to detect luciferase activity using Dual-luciferase Reporter Gene Assay Kit (Beyotime).

### Xenograft models

U2OS cells transfected with sh-NC or sh-circ_0000376 were subcutaneously injected into BALB/c nude mice (6-week-old, Vital River, Beijing, China) to construct xenograft tumor model (n = 6/group). Tumor volume was recorded every 3 days post-injection 7 days. 22 days later, tumor tissues were excised from euthanized mice. Mice tumor tissues were used for preparing paraffin section. Immunohistochemical (IHC) staining was carried out using SP Kit (Solarbio) with anti-HK2 (1:500, ab227198), anti-LDHA (1:2000, ab52488) and anti-Ki67 (1:1000, ab15580). Animal experiment was approved by The Third Hospital of Mianyang.

### Statistical analysis

Data were shown as means ± SD. GraphPad Prism 7.0 was used to perform statistical analyses. Significant differences were compared using Student’s *t*-test or ANOVA. *P* < 0.05 was considered statistically significant.

## Results

### Circ_0000376 expression was increased in OS patients and cells

**Fig. 1:**
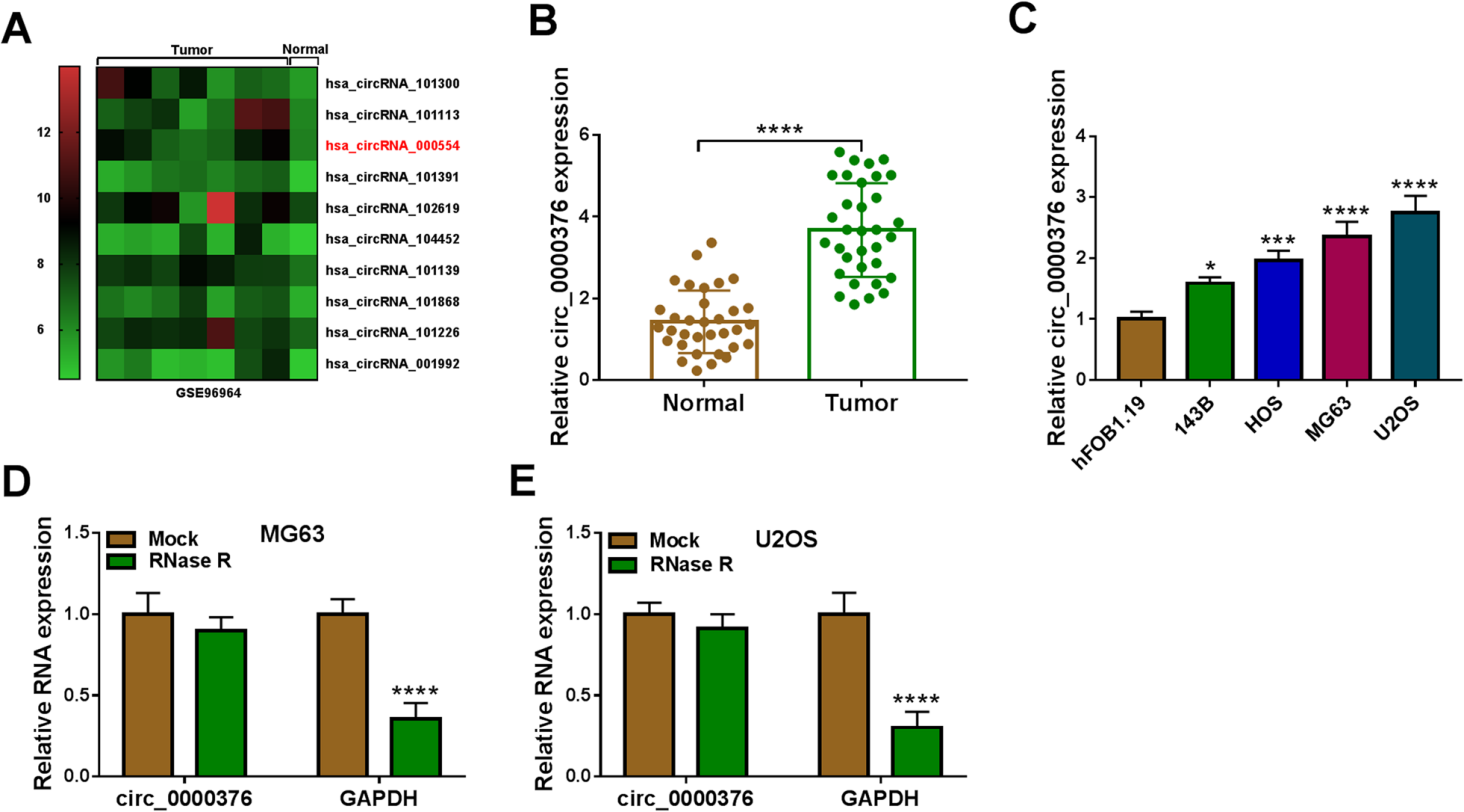
Circ_0000376 expression in OS patients and cells. **A** Heat map showed differentially expressed circRNA in OS tumor tissues and normal tissues in GSE96964. **B** Circ_0000376 expression in 33 paired OS tumor tissues and adjacent normal tissues was examined by qRT-PCR. **C** Circ_0000376 expression in OS cells and hFOB1.19 cells was detected by qRT-PCR. **D**, **E** After treated with RNase R, circ_0000376 and linear RNA GAPDH expression was determined by qRT-PCR. *P < 0.05, ***P < 0.001, ****P < 0.0001

Figure 1A exhibited 10 differentially expressed circRNAs in OS tumor tissues and normal tissues in GEO database (accession: GSE96964), among which circ_0000376 (chip: hsa_circRNA_000554) was significantly overexpressed in OS tumor tissues. Through qRT-PCR, circ_0000376 was confirmed to be upregulated in OS tumor tissues compared to adjacent normal tissues (Fig. 1B), as well as in 4 OS cell lines compared to hFOB1.19 cells (Fig. 1C). After RNA was treated with RNase R, we confirmed that circ_0000376 expression was not significantly affected, while linear RNA GAPDH mRNA expression was markedly reduced (Fig. 1D, E). These data confirmed that circ_0000376 could resist RNA digestion.

### Knockdown of circ_0000376 inhibited OS cell growth, invasion and glycolysis

**Fig. 2:**
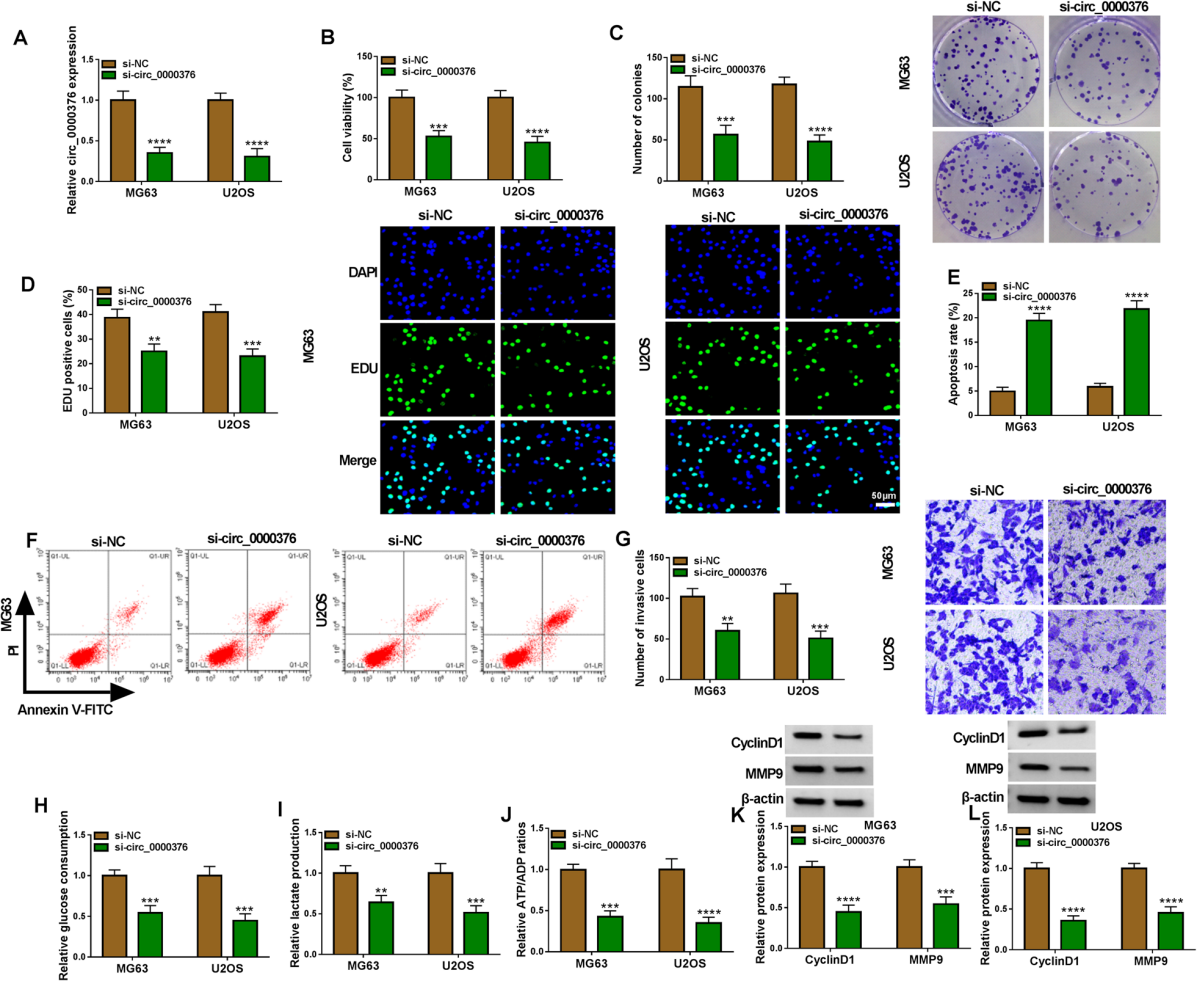
Effects of si-circ_0000376 on OS cell progression. MG63 and U2OS cells were transfected with si-NC and si-circ_0000376. **A** The circ_0000376 expression was evaluated by qRT-PCR. CCK8 assay (**B**), colony formation assay (**C**), EDU assay (**D**), flow cytometry (**E**, **F**) and transwell assay (**G**) were used to measure cell proliferation, apoptosis and invasion. **H**–**J** Glucose consumption, lactate production and ATP/ADP ratio were determined to measure cell glycolysis. **K**, **L** The protein levels of CyclinD1 and MMP9 were tested by WB analysis. **P < 0.01, ***P < 0.001, ****P < 0.0001

After si-circ_0000376 was transfected into MG63 and U2OS cells, circ_0000376 expression was remarkably decreased (Fig. 2A). Then, we evaluated OS cell proliferation, apoptosis, invasion and glycolysis to explore the effect of circ_0000376 knockdown on OS cell progression. As shown in Fig. 2B–F, downregulation of circ_0000376 suppressed cell viability, the number of colonies and EDU positive cell rate, while increased cell apoptosis rate. Additionally, circ_0000376 knockdown inhibited the number of invasive cells, glucose consumption, lactate production and ATP/ADP ratios (Fig. 2G–J). Moreover, circ_0000376 knockdown resulted in a decrease in ECAR and an increase in OCR in MG63 cells (Additional file 1: Fig. S1A-B), confirming that circ_0000376 might promote Warburg effect of OS cells. The results exhibited that. WB analysis results indicated that silencing of circ_0000376 also decreased cell cycle protein CyclinD1 expression and invasion protein MMP9 expression in OS cells (Fig. 2K–L). These results indicated that circ_0000376 enhanced OS cell proliferation, invasion, glycolysis and inhibited apoptosis.

### Circ_0000376 interacted with miR-577

**Fig. 3:**
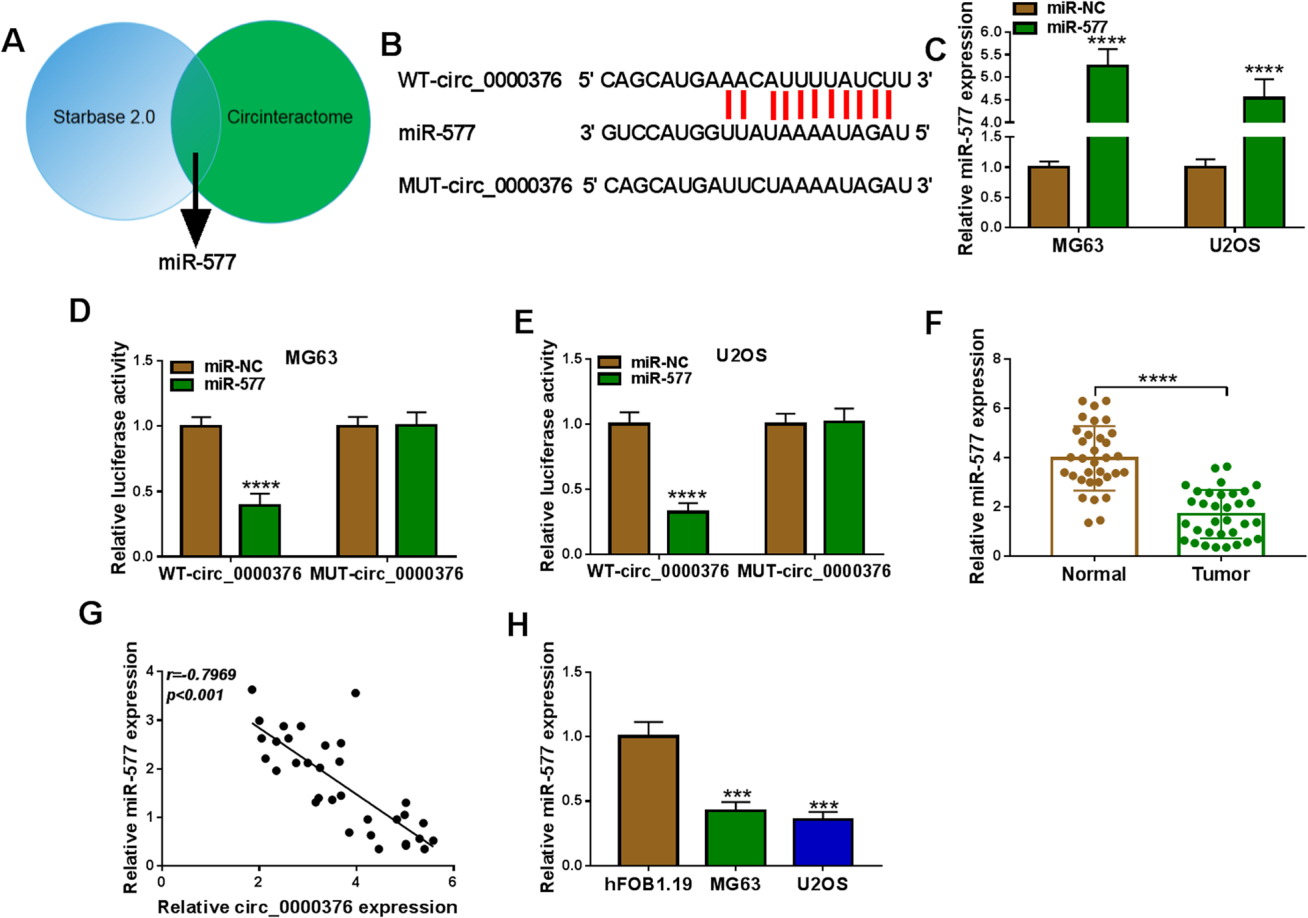
Circ_0000376 sponged miR-577. **A** Venn diagram showed the miRNA predicted by starbase software and circinteractome software together. **B** The binding sites between circ_0000376 and miR-577 were exhibited. **C** The transfection efficiency of miR-577 mimic was assessed by qRT-PCR. **D**, **E** Dual-luciferase reporter assay was used to confirm the interaction between circ_0000376 and miR-577. **F** MiR-577 expression was examined by qRT-PCR in 33 paired OS tumor tissues and adjacent normal tissues. **G** Pearson correlation analysis was used. **H** MiR-577 expression was detected by qRT-PCR in OS cells and hFOB1.19 cells. **P < 0.01, ***P < 0.001, ****P < 0.0001

The starbase software and circinteractome software were used to jointly predict miRNAs that could complement with circ_0000376, and then we focused on miR-577 (Fig. 3A). According to their binding sites, we designed the WT/MUT-circ_0000376 reporter vectors (Fig. 3B). Besides, miR-577 mimic was used to overexpress miR-577 in MG63 and U2OS cells (Fig. 3C). In dual-luciferase reporter assay, we observed that the luciferase activity of WT-circ_0000376 vector without MUT-circ_0000376 vector was reduced by miR-577 mimic, confirming the interaction between circ_0000376 and miR-577 (Fig. 3D, E). In OS tumor tissues, miR-577 had decreased expression and was negatively correlated with circ_0000376 expression (Fig. 3F–G). Also, miR-577 was lowly expressed in OS cells (MG63 and U2OS) compared to hFOB1.19 cells (Fig. 3H). Above data confirmed that circ_0000376 could sponge miR-577.

### The regulation of si-circ_0000376 on OS cell progression was eliminated by anti-miR-577

**Fig. 4:**
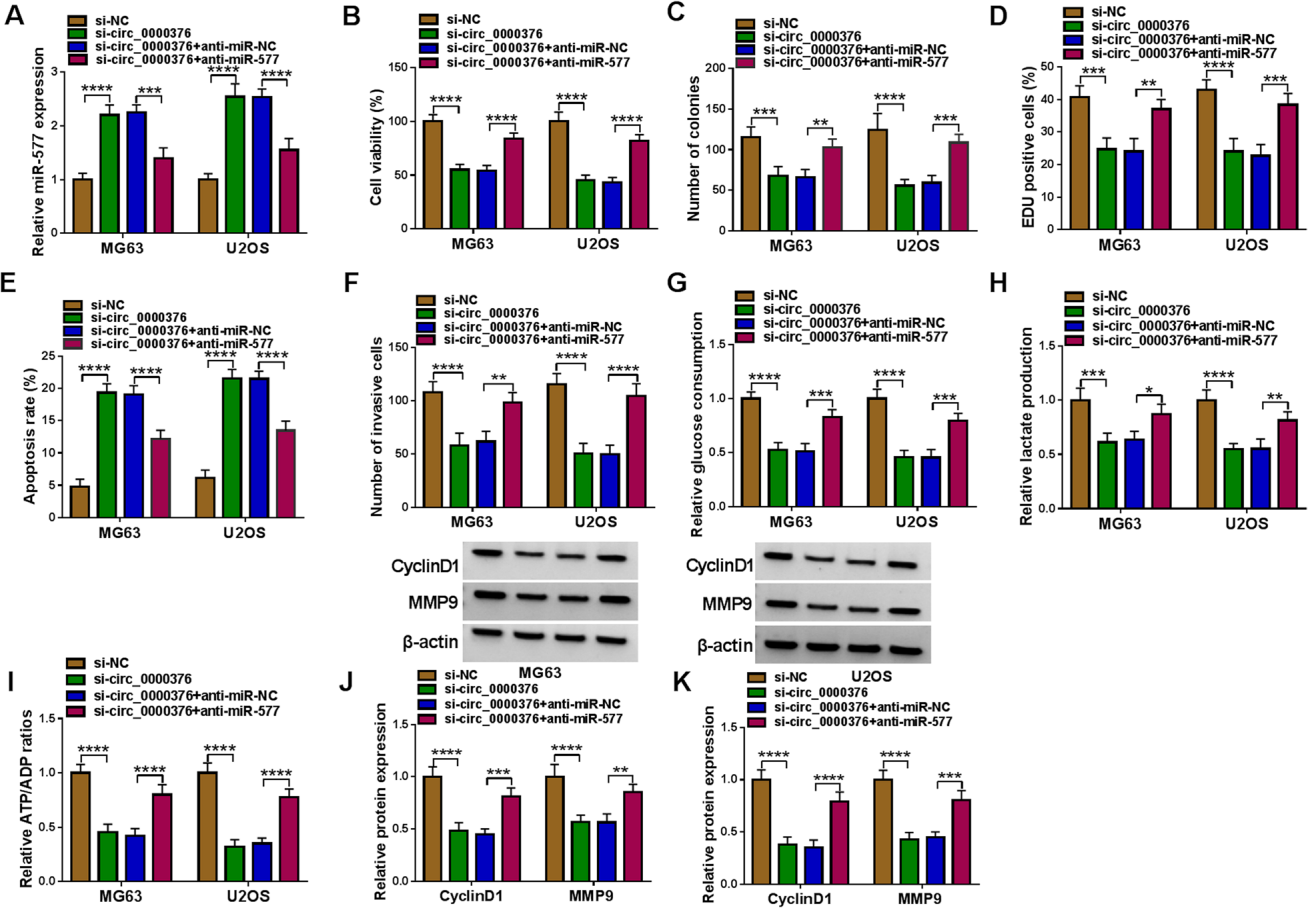
Effects of si-circ_0000376 and anti-miR-577 on OS cell progression. MG63 and U2OS cells were transfected with si-circ_0000376 and anti-miR-577. **A** The miR-577 expression was detected by qRT-PCR. Cell proliferation, apoptosis and invasion were determined using CCK8 assay (**B**), colony formation assay (**C**), EDU assay (**D**), flow cytometry (**E**) and transwell assay (**F**). **G**–**I** Cell glycolysis was assessed by glucose consumption, lactate production and ATP/ADP ratio. **J**, **K** WB analysis was used to measure the protein levels of CyclinD1 and MMP9. *P < 0.05, **P < 0.01, ***P < 0.001, ****P < 0.0001

To explore whether circ_0000376 regulated OS progression via sponging miR-577, the rescue experiments were performed. After co-transfected with si-circ_0000376 and anti-miR-577 into MG63 and U2OS cells, we detected miR-577 expression and confirmed that miR-577 expression promoted by si-circ_0000376 could be decreased by anti-miR-577 (Fig. 4A). Analysis results showed that the negative regulation of si-circ_0000376 on cell viability, the number of colonies and EDU positive cell rate were reversed by miR-577 inhibitor (Fig. 4B–D and Additional file 2: Fig. S2A-B). Circ_0000376 knockdown induced cell apoptosis could also be abolished by miR-577 inhibitor (Fig. 4E and Additional file 2: Fig. S2C). Furthermore, the addition of anti-miR-577 overturned the suppressive effects of si-circ_0000376 on the number of invasive cells, glucose consumption, lactate production, ATP/ADP ratio, and the protein expression of CyclinD1 and MMP9 (Fig. 4F–K and Additional file 2: Fig. S2D). Therefore, we confirmed that circ_0000376 might contribute to OS progression via targeting miR-577.

### MiR-577 interacted with HK2 and LDHA

**Fig. 5:**
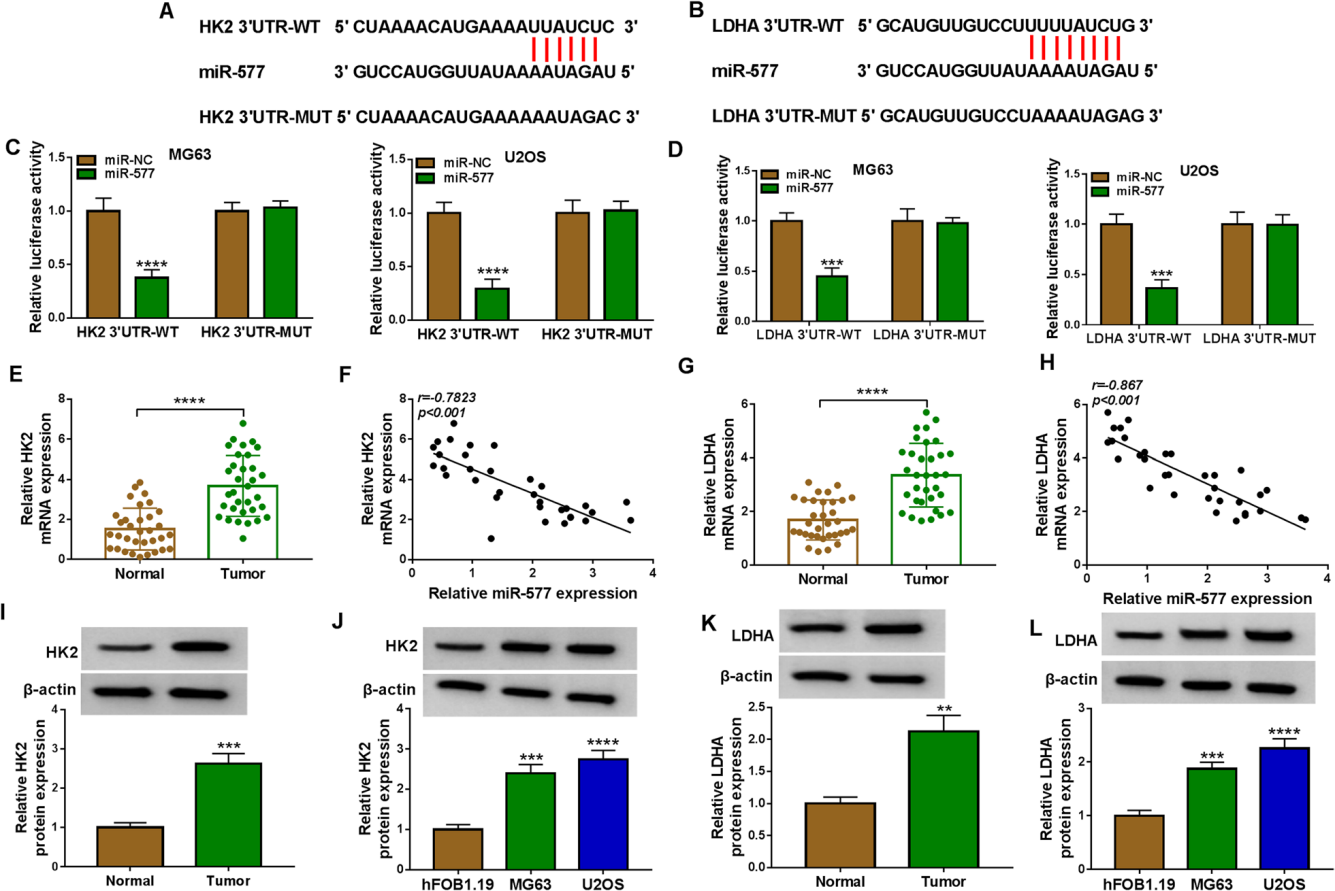
MiR-577 targeted HK2 and LDHA. **A**, **B** The binding sites between miR-577 and HK2 3’UTR or LDHA 3’UTR were exhibited. **C**, **D** Dual-luciferase reporter assay was used to confirm the interaction between miR-577 and HK2 or LDHA. **E** HK2 mRNA expression in 33 paired OS tumor tissues and adjacent normal tissues was determined by qRT-PCR. **F** Pearson correlation analysis was performed. **G** QRT-PCR was used to detect LDHA mRNA expression in 33 paired OS tumor tissues and adjacent normal tissues. **H** Pearson correlation analysis was used. **I**–**L** HK2 and LDHA protein expression levels in OS tissues and cells were detected by WB analysis. **P < 0.01, ***P < 0.001, ****P < 0.0001

Targetscan software was used to predict the downstream target of miR-577. The 3’UTRs of HK2 and LDHA were discovered to have binding sites with miR-577 (Fig. 5A, B). MiR-577 mimic reduced the luciferase activities of the HK2 3’UTR-WT vector and LDHA 3’UTR-WT vector, confirmed that there had interaction relationship between miR-577 and HK2 or LDHA (Fig. 5C, D). HK2 and LDHA mRNA expression levels were upregulated in OS tumor tissues, and their expression levels were negatively correlated with miR-577 expression (Fig. 5E–H). In OS tumor tissues and cells, we also observed the high HK2 and LDHA expression at the protein levels (Fig. 5I–L).

### MiR-577 hindered OS cell progression by targeting HK2 and LDHA

**Fig. 6:**
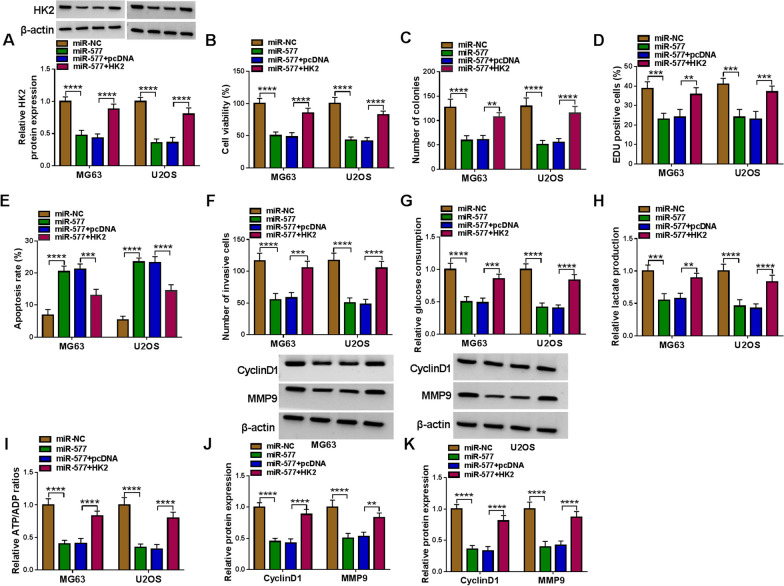
Effects of miR-577 and HK2 on OS cell progression. MG63 and U2OS cells were transfected with miR-577 mimic and pcDNA HK2 overexpression vector. **A** HK2 protein expression was detected by WB analysis. CCK8 assay (**B**), colony formation assay (**C**), EDU assay (**D**), flow cytometry (**E**) and transwell assay (**F**) were employed to analyze cell proliferation, apoptosis and invasion. **G**–**I** Glucose consumption, lactate production and ATP/ADP ratio were analyzed to measure cell glycolysis. **J**–**K** WB analysis was performed to detect CyclinD1 and MMP9 protein levels. **P < 0.01, ***P < 0.001, ****P < 0.0001

**Fig. 7:**
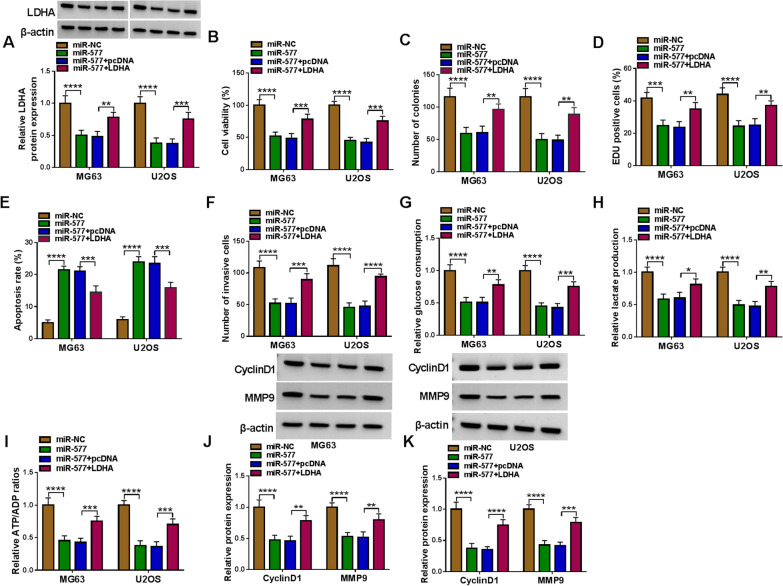
Effects of miR-577 and LDHA on OS cell progression. MG63 and U2OS cells were transfected with miR-577 mimic and pcDNA LDHA overexpression vector. **A** The LDHA protein expression was tested using WB analysis. Cell proliferation, apoptosis and invasion were analyzed by CCK8 assay (**B**), colony formation assay (**C**), EDU assay (**D**), flow cytometry (**E**) and transwell assay (**F**). **G**–**I** Glucose consumption, lactate production and ATP/ADP ratio were examined to evaluate cell glycolysis. **J**–**K** The protein levels of CyclinD1 and MMP9 were analyzed using WB analysis. *P < 0.05, **P < 0.01, ***P < 0.001, ****P < 0.0001

To further confirm that miR-577 mediated OS progression by regulating HK2 and LDHA, we conducted rescue tests, respectively. In MG63 and U2OS cells co-transfected with miR-577 mimic and pcDNA HK2 overexpression vector, we found that miR-577 reduced HK2 protein expression, and this effect was reversed by pcDNA HK2 overexpression vector (Fig. 6A). MiR-577 inhibited cell viability, the number of colonies and EDU positive cell rate, while enhanced apoptosis rate. However, these effects were reversed by HK2 overexpression (Fig. 6B–E and Additional file 3: Fig. S3A-C). Moreover, overexpressed HK2 also eliminated the inhibitory effects of miR-577 on the number of invasive cells, glucose consumption, lactate production, ATP/ADP ratio, and the protein expression of CyclinD1 and MMP9 (Fig. 6F–K and Additional file 3: Fig. S3D). Similarly, pcDNA LDHA overexpression vector were transfected into MG63 and U2OS cells with miR-577 mimic. As shown in Fig. 7A, pcDNA LDHA overexpression vector increased LDHA protein expression reduced by miR-577. Function experiments suggested that LDHA overexpression overturned the regulation of miR-577 on OS cell proliferation, apoptosis, invasion, and glycolysis (Fig. 7B–I and Additional file 4: Fig. S4A-D). Also, the decreasing effect of miR-577 on the protein expression of CyclinD1 and MMP9 was abolished by overexpressing LDHA (Fig. 7J, K). Above all, these results suggested that miR-577 targeted HK2/LDHA to suppress OS progression.

### Interference of circ_0000376 inhibited OS tumor growth

**Fig. 8:**
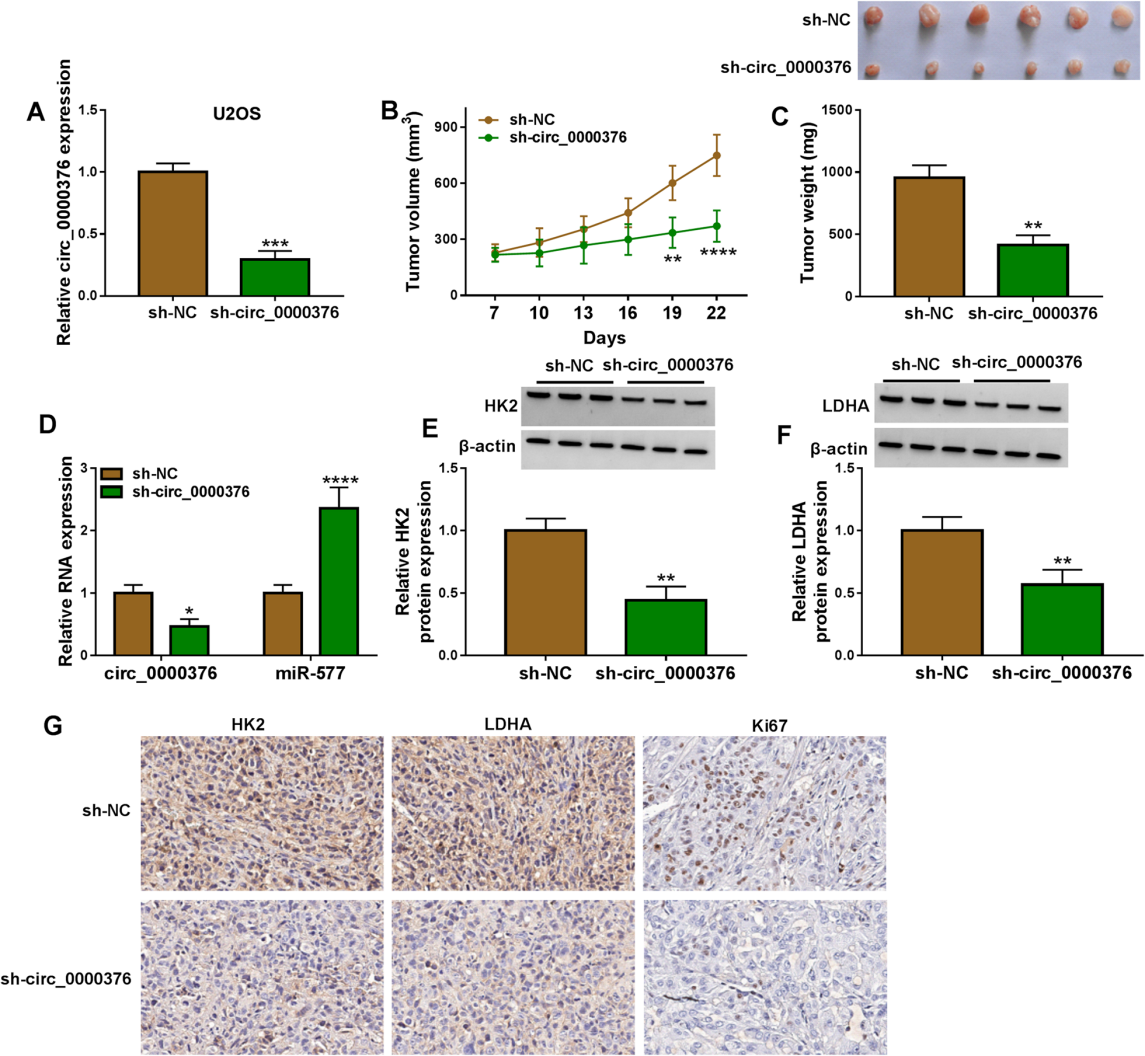
Effects of sh-circ_0000376 on OS tumor growth. **A** Circ_0000376 expression was detected by qRT-PCR in U2OS cells transfected with sh-NC or sh-circ_0000376.** B**–**G** U2OS cells transfected with sh-NC or sh-circ_0000376 were injected into nude mice. Tumor volume (**B**) and weight (**C**) were measured. **D** QRT-PCR was used to detect circ_0000376 and miR-577 expression. **E**, **F** WB analysis was performed to determine HK2 and LDHA protein levels. **G** IHC staining was used to determine HK2, LDHA and Ki67 positive cells. *P < 0.05, **P < 0.01, ***P < 0.001, ****P < 0.0001

To determine the role of circ_0000376 in vivo, we constructed U2OS cells with stable knockdown circ_0000376 using sh-circ_0000376 (Fig. 8A). After that, U2OS cells transfected with sh-NC/sh-circ_0000376 were injected into nude mice. After 22 days, we found that tumor volume and weight were reduced in the sh-circ_0000376 group (Fig. 8B, C). In the mice tumor tissues of sh-circ_0000376 group, circ_0000375 expression was inhibited and miR-577 expression was promoted (Fig. 8D). Also, The HK2 and LDHA protein expression levels were repressed in sh-circ_0000376 group (Fig. 8E, F). Besides, HK2, LDHA and Ki67 positive cells also were decreased in the tumor tissues of sh-circ_0000376 group (Fig. 8G). These results showed that circ_0000376 sponged miR-577 to promote HK2/LDHA-mediated glycolysis, thus accelerating OS tumor growth in vivo.

## Discussion

Circ_0000376 acts as an oncogenic gene in many tumors. For example, circ_0000376 was considered to be a tumor promoter in lung cancer, which enhanced lung cancer proliferation, glycolysis and metastasis through miRNA/mRNA network [18–20]. Also, circ_0000376 had been shown to play active role the malignant progression of gastric cancer and breast cancer [21, 22]. Here, we investigated circ_0000376 role in OS. The present results suggested that circ_0000376 was overexpressed in OS, and its interference restrained OS cell proliferation, invasion, glycolysis, and accelerated apoptosis. Animal experiments also further showed that circ_0000376 knockdown reduced OS tumorigenesis in vivo. These results provided new evidence that circ_0000376 was a potential therapeutic target for OS. We believed that circ_0000376 promoted OS malignant progression, which was consistent with the previous reports [17].

MiRNA and siRNAs have been confirmed to play vital function in human diseases [23–27]. According to reported studies, circ_0000376 might be involved in regulating OS development through sponging miR-432-5p [17]. Here, we explored the new molecular mechanism of circ_0000376, and confirmed that circ_0000376 sponged miR-577. In many tumors, miR-577 played a negative role in tumor malignant phenotype, such as breast cancer [28] and glioblastoma [29]. MiR-577 suppressed the proliferation and metastasis of papillary thyroid carcinoma cells [30], and could inhibit cervical cancer cell growth and glycolysis [31]. In the previous research, miR-577 had been discovered to be lowly expressed in OS, which could reduce OS proliferation and migration [32]. Similar to this reports, we also found that miR-577 had the ability to inhibit OS progression in this study. In functional experiments, miR-577 suppressed OS cell growth, invasion and glycolysis. Circ_0000376 negatively regulated miR-577 level, and miR-577 inhibitor also revoked si-circ_0000376-mediated OS cell function. These results provided evidence that circ_0000376 targeted miR-577 to regulate OS progression.

Glycolysis is one of the prominent features of malignant tumors and is the main source of energy during tumor growth [33, 34]. HK2, a member of HK family, is a key rate-limiting enzyme in glycolysis pathway, mainly responsible for catalyzing glucose phosphorylation [35]. LDHA is also a key enzyme in the glycolysis pathway that converts pyruvate to lactic acid [36]. Many studies had confirmed that the increased expression of HK2 and LDHA promoted the glycolysis process of tumor cells, thus accelerating the malignant phenotype of tumors, such as hepatocellular carcinoma [37] and bladder cancer [38]. Research had suggested that HK2 was overexpressed in OS, and its overexpression promoted OS cell proliferation and invasion [39, 40]. Besides, LDHA had been shown to be upregulated in OS, which enhanced cell growth and metastasis to promote OS progression [41, 42]. Here, we pointed out that miR-577 targeted HK2 and LDHA. Overexpressed HK2 and LDHA reversed miR-577-mediated the inhibition on OS cell growth, invasion and glycolysis, confirming that miR-577 indeed suppressed OS development through targeting HK2 and LDHA. Importantly, circ_0000376 had a positively regulation on HK2 and LDHA expression, which perfected the mechanism of circ_0000376/miR-577/HK2/LDHA axis.

**Fig. 9:**
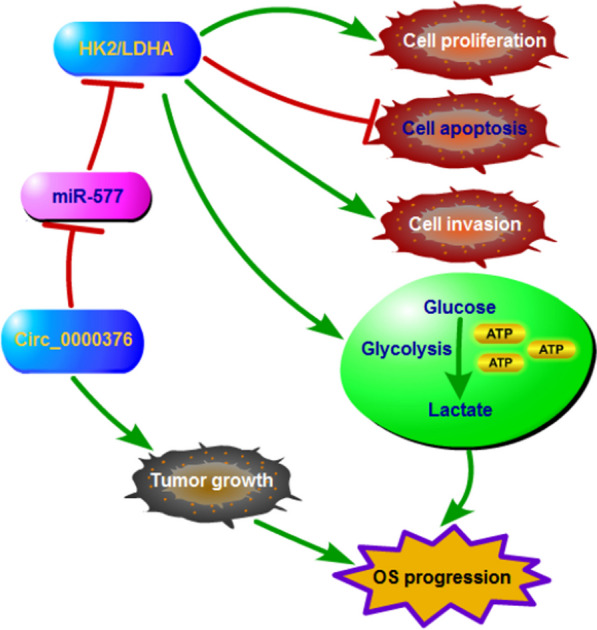
Mechanism diagram of this study. Circ_0000376 promoted OS cell proliferation, invasion, glycolysis and inhibited apoptosis by regulating miR-577/HK2/LDHA axis

In summary, we provided strong evidence that circ_0000376 played a key role in OS development, which promoted OS growth, invasion and glycolysis through miR-577/HK2/LDHA pathway (Fig. 9). Inhibition of circ_0000376 might be an effective treatment method for OS, offering new evidence that circ_0000376 served as a potential therapeutic target for OS.

### Supplementary Information

Below is the link to the electronic supplementary material.

**Additional file 1: Fig. S1.** Effect of si-circ_0000376 on ECAR and OCR of OS cells. were transfected with si-NC and si-circ_0000376. An XF96 extracellular flux analyzer was employed to analyze the ECAR (A) and OCR (B) of MG63 cells.

**Additional file 2: Fig. S2**. The representative pictures of Fig. 4C (A), 4D (B), 4E (C), and 4F (D).**P< 0.01, ***P < 0.001, ****P < 0.0001.

**Additional file 3: Fig. S3**. The representative pictures of Fig. 6C (A), 6D (B), 6E (C), and 6F (D).**P< 0.01, ***P < 0.001, ****P < 0.0001.

**Additional file 4: Fig. S4.** The representative pictures of Fig. 7C (A), 7D (B), 7E (C), and 7F (D).**P< 0.01, ***P < 0.001, ****P < 0.0001.

## Data Availability

The analyzed data sets generated during the present study are available from the corresponding author on reasonable request.
