# Supplementary figures and images for: Circ_0000376 regulates miR-577/HK2/LDHA signaling pathway to promote the growth, invasion and glycolysis of osteosarcoma
Source: J Orthop Surg Res. 2024 Jan 13;19:67. doi: 10.1186/s13018-023-04520-y (PMC10788008; doi:10.1186/s13018-023-04520-y)

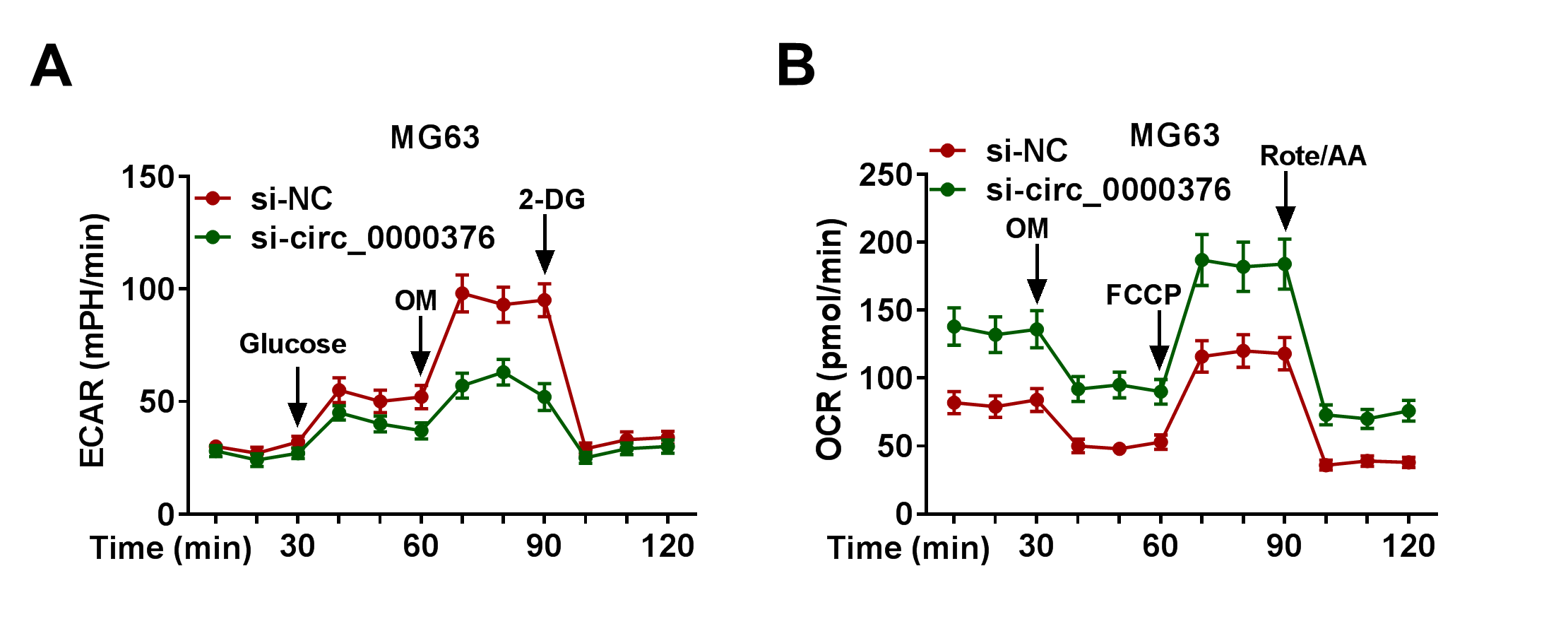

Supplement: Supplementary file 1 — Additional file 1: Fig. S1. Effect of si-circ_0000376 on ECAR and OCR of OS cells. were transfected with si-NC and si-circ_0000376. An XF96 extracellular flux analyzer was employed to analyze the ECAR (A) and OCR (B) of MG63 cells. [file 13018_2023_4520_MOESM1_ESM.tif]

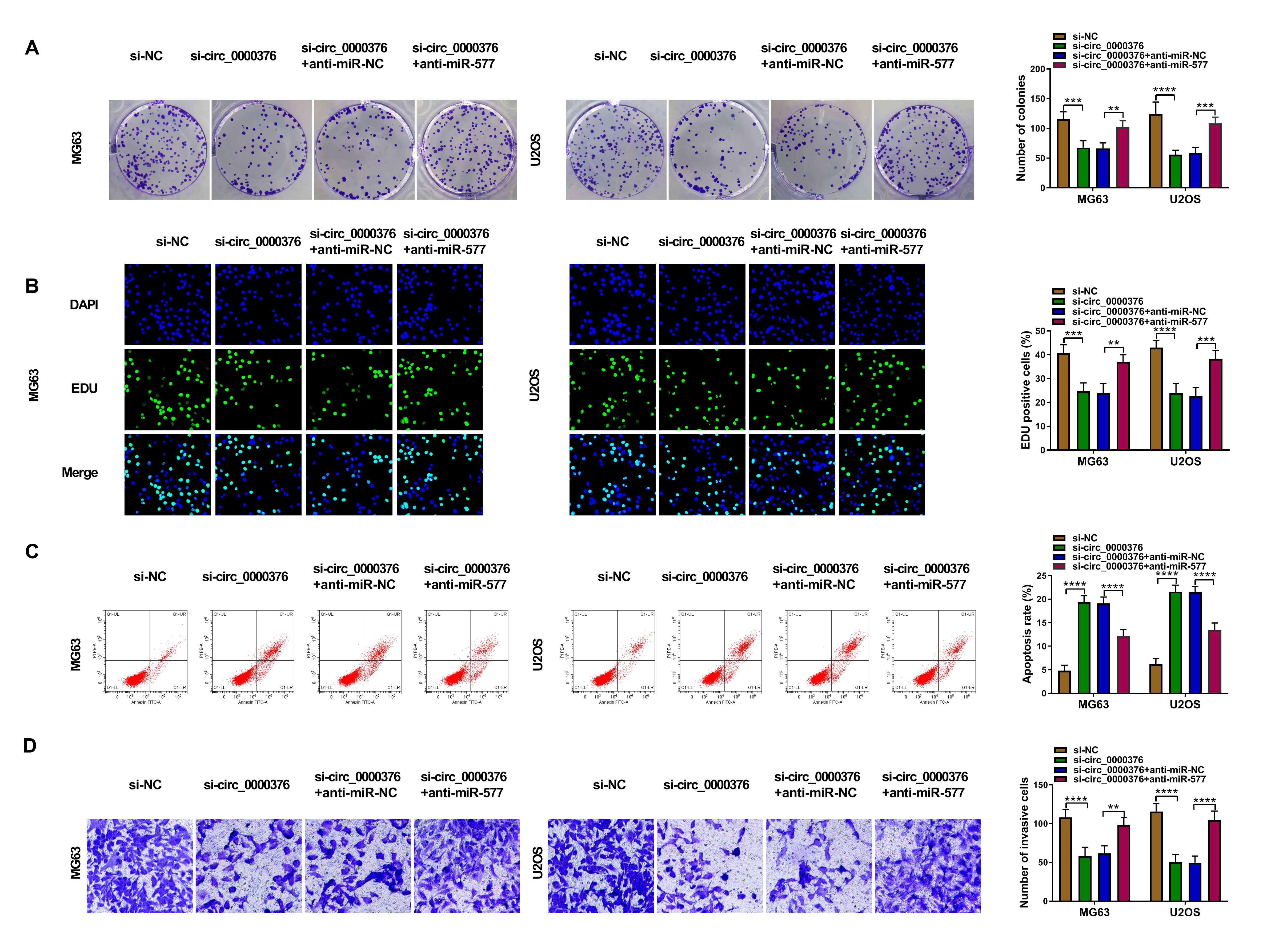

Supplement: Supplementary file 2 — Additional file 2: Fig. S2. The representative pictures of Fig. 4C (A), 4D (B), 4E (C), and 4F (D).**P< 0.01, ***P < 0.001, ****P < 0.0001. [file 13018_2023_4520_MOESM2_ESM.tif]

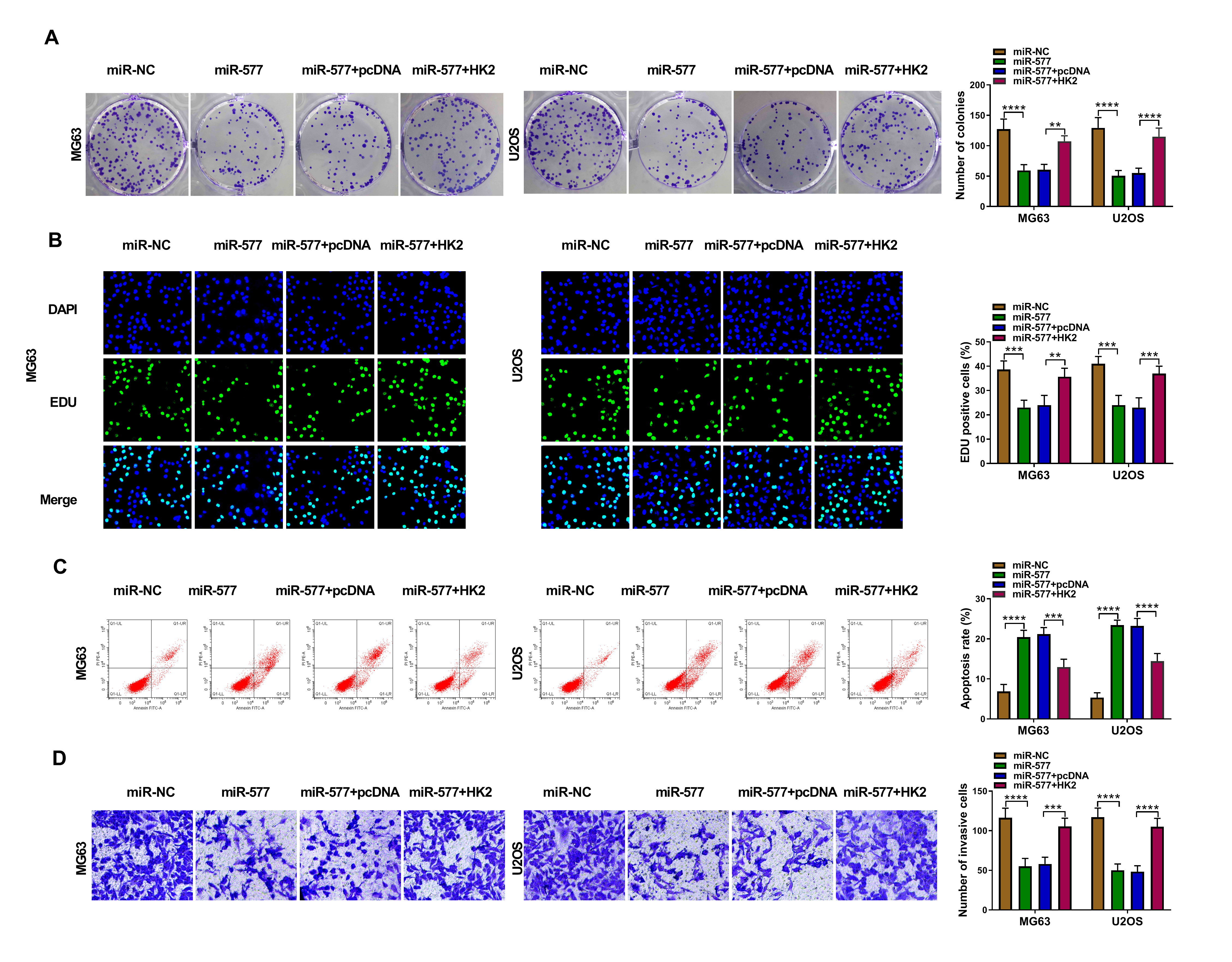

Supplement: Supplementary file 3 — Additional file 3: Fig. S3. The representative pictures of Fig. 6C (A), 6D (B), 6E (C), and 6F (D).**P< 0.01, ***P < 0.001, ****P < 0.0001. [file 13018_2023_4520_MOESM3_ESM.tif]

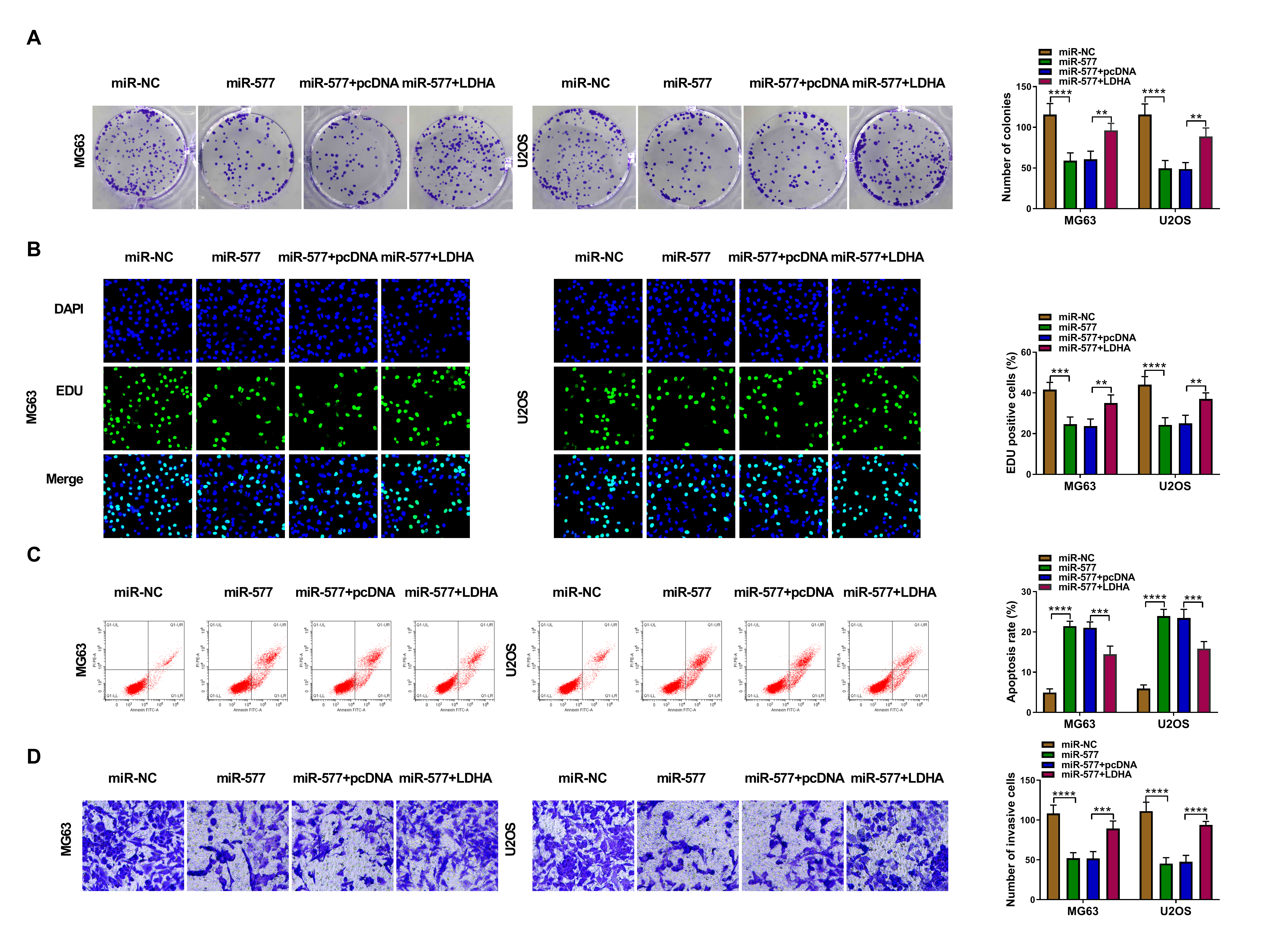

Supplement: Supplementary file 4 — Additional file 4: Fig. S4. The representative pictures of Fig. 7C (A), 7D (B), 7E (C), and 7F (D).**P< 0.01, ***P < 0.001, ****P < 0.0001. [file 13018_2023_4520_MOESM4_ESM.tif]
